# The COVID-19 pandemic and new clinical trial activations

**DOI:** 10.1186/s13063-021-05219-3

**Published:** 2021-04-08

**Authors:** Joseph M. Unger, Hong Xiao

**Affiliations:** Fred Hutchinson Cancer Research Center, 1100 Fairview Ave N, M3-C102, PO Box 19024, Seattle, WA 98109-1024 USA

**Keywords:** COVID-19, Cancer clinical trials, Cardiovascular clinical trials, Mental health clinical trials, ClinicalTrials.gov

## Abstract

**Background:**

The COVID-19 pandemic has caused severe disruptions in care for many patients. A key question is whether the landscape of clinical research has also changed.

**Methods:**

In a retrospective cohort study, we examined the association of the COVID-19 outbreak with new clinical trial activations. Trial data for all interventional and observational oncology, cardiovascular, and mental health studies from January 2015 through September 2020 were obtained from ClinicalTrials.gov. An interrupted time-series analysis with Poisson regression was used.

**Results:**

We examined 62,252 trial activations. During the initial COVID-19 outbreak (February 2020 through May 2020), model-estimated monthly trial activations for US-based studies were only 57% of the expected estimate had the pandemic not occurred (relative risk = 0.57, 95% CI 0.52 to 0.61, *p* < .001). For non-US-based studies, the impact of the pandemic was less dramatic (relative risk = 0.77, 95% CI 0.73 to 0.82, *p* < .001), resulting in an overall 27% reduction in the relative risk of new trial activations for US-based trials compared to non-US-based trials (relative risk ratio = 0.73, 95% CI 0.67 to 0.81, *p* < .001). Although a rebound occurred in the initial reopening phase (June 2020 through September 2020), the rebound was weaker for US-based studies compared to non-US-based studies (relative risk ratio = 0.87, 95% CI 0.80 to 0.95, *p* < .001).

**Conclusions:**

These findings are consistent with the disproportionate burden of COVID-19 diagnoses and deaths during the initial phase of the pandemic in the USA. Reduced activation of cancer clinical trials will likely slow the pace of clinical research and new drug discovery, with long-term negative consequences for cancer patients. An important question is whether the renewed outbreak period of winter 2020/2021 will have a similarly negative impact on the initiation of new clinical research studies for non-COVID-19 diseases.

## Background/aims

The COVID-19 pandemic has caused severe interruptions in care for patients with non-COVID-19 diseases [1]. As research into COVID-19 treatments has dramatically increased, a key question is whether the landscape of clinical research for non-COVID-19 diseases has also changed, especially in the USA, where a disproportionate number of cases and deaths from COVID-19 have occurred [2]. This is important since advances in new treatments for patients rely on the conduct of clinical trials, which represent a key final step in proving the efficacy of new therapies. Thus interruptions in the conduct of clinical trial research could slow the development of new treatments for common illnesses.

Our aim was to examine the association of the COVID-19 outbreak with new clinical trial activations.

## Methods

To address this, in a retrospective cohort study, we examined the association of the COVID-19 outbreak with new clinical trial activations using data from ClinicalTrials.gov, a comprehensive catalog of all registered domestic and international clinical trials [3]. All interventional and observational oncology, cardiovascular, and mental-health studies—the most common clinical research categories [3]—were included. Poisson regression was used to examine whether total monthly trial activations (using study activation date) changed over time. The World Health Organization declared a Public Health Emergency of International Concern on January 30, 2020. Using an interrupted time-series approach, the exposures were landmark timepoint indicator variables describing whether trial activations occurred in (1) the initial pandemic period (February–May 2020) versus the pre-pandemic period (2015–January 2020); and (2) the initial US-based reopening period (June–September 2020) versus before June 2020. Comparisons between US-based and non-US-based activations were conducted using interaction tests. The pre-pandemic period was established over 5 years to better model monthly variation. We also included indicator variables for calendar month, with January as the reference, to account for seasonal variation (especially the bolus of trial activations typically observed in January) and potential temporal autocorrelation.

We also evaluated the extent to which the estimated number of trials not activated during the initial pandemic period were “replaced” by the excess number of trials activated in the reopening period. COVID-19 trial activations over time were described.

We followed the STROBE reporting guideline for cohort studies [4]. This study was exempt from institutional review board oversight given no patient-level data. Analyses were conducted in R-version-4.0.2 (R-Project for Statistical Computing) using data obtained November 17, 2020. A 2-sided *P* < .05 indicated statistical significance.

## Results

Overall, 62,252 trial activations were examined. Non-COVID-19 trial activations (*n* = 58,888; 21,684 US, 37,204 non-US) included oncology (*n* = 29,336, 49.8%), cardiovascular (*n* = 16,239, 27.5%), mental-health (*n* = 11,283, 19.2%), and multiple (*n* = 2030, 3.4%) disease studies. More studies were industry-sponsored (16,022, 27.2%) versus government-sponsored (4869, 8.3%). Among studies with known data, most were treatment trials (31,129/45,430 = 68.5%) evaluating systemic therapies (26,492/54,180 = 48.9%). COVID-19 activations included *n* = 3364 studies (3364/62,252 = 5.4%; 725 US; 2639 non-US).

**Fig. 1:**
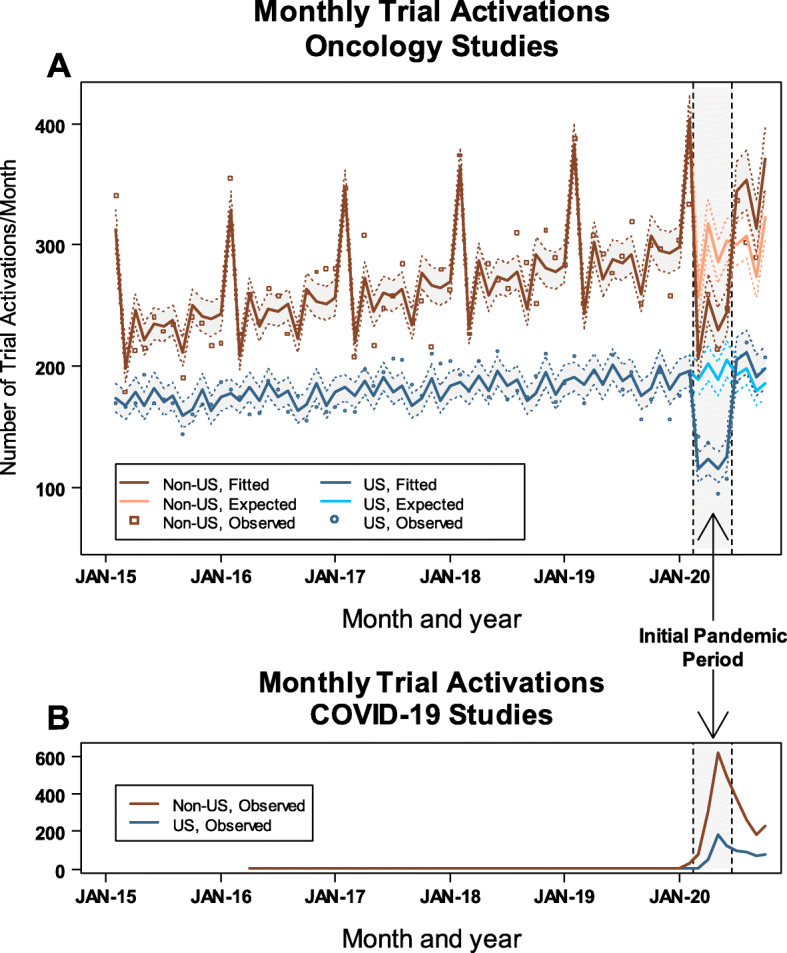
Monthly trial activations over time. **a** Monthly trial activations for oncology, cardiovascular, and mental-health studies. The gray rectangular shaded area represents the initial pandemic period. The red squares and blue circles show observed non-US-based and US-based (respectively) monthly trial activation totals. The dark red line indicates the Poisson model-fitted (or predicted) non-US-based monthly activation totals; the lighter orange shows the model-based expected (or counterfactual) number of activations had the pandemic not occurred. Similarly, the dark blue line indicates the Poisson regression model-fitted US-based monthly activation totals, and the lighter blue line shows the model-based expected number of activations had the pandemic not occurred. Panel B) Observed monthly trial activations for COVID-19 studies. The red and blue lines show the observed number of trial activations for non-US-based and US-based studies, respectively

In the USA, there were fewer trial activations for non-COVID-diseases during the initial pandemic period (Fig. 1a), as the number of COVID-19 trials increased dramatically (Fig. 1b). Model-estimated monthly trial activations were only 57% (201.5; relative risk = 0.57, 95% CI 0.52 to 0.61, *p* < .001) of the expected (i.e., counterfactual) estimate had the pandemic not occurred (355.6/month; Table 1). In contrast, for non-US-based trials, the decrease in monthly trial activations during the pandemic period was more modest, with model-estimated monthly trial activations 77% (437.0/month) of the expected estimate had the pandemic not occurred (565.9/month; relative risk = 0.77, 95% CI 0.73 to 0.82, *p* < .001). Thus overall, there was a 27% reduction in the relative risk of new trial activations for US-based trials compared to non-US-based trials (relative risk ratio = 0.73, 95% CI 0.67–0.81, *p* < .001; Table 1).

**Table 1:** Comparison of estimated relative change in monthly number of trials in US and non-US regions

**Initial pandemic period**										
		Monthly trial registrations^1^			Relative risk (RR)^1^			RR ratio (US versus non-US)^2^		
Trial type	Region	Fitted value (*n*) 2015–Jan 2020	Fitted value (*n*) Feb–May 2020	Expected value^3^ Feb–May 2020	RR	95% CI	*p* value	Risk ratio	95% CI	*p* value
All trials	US	320.1 (*n* = 19,524)	201.5 (*n* = 806)	355.6	0.57	0.52, 0.61	< .001	0.73	0.67, 0.81	< .001
	Non-US	539.0 (*n* = 32,876)	437.0 (*n* = 1748)	565.9	0.77	0.73 0.82	< .001			
Oncology	US	180.8 (*n* = 11,028)	120.5 (*n* = 482)	196.7	0.61	0.55, 0.68	< .001	0.76	0.67, 0.87	< .001
	Non-US	267.9 (*n* = 16, 344)	234.0 (*n* = 936)	290.9	0.80	0.75, 0.87	< .001			
Cardiovascular	US	75.6 (*n* = 4, 609)	44.0 (*n* = 176)	80.6	0.55	0.46, 0.65	< .001	0.75	0.61, 0.90	.003
	Non-US	189.5 (*n* = 11, 558)	135.3 (*n* = 541)	184.6	0.73	0.66, 0.81	< .001			
Mental health	US	77.3 (*n* = 4, 713)	46.6 (*n* = 186)	93.8	0.50	0.42, 0.58	< .001	0.69	0.56, 0.84	< .001
	Non-US	97.6 (*n* = 5, 955)	81.3 (*n* = 325)	112.3	0.72	0.64, 0.82	< .001			
**Reopening period**										
			Fitted value (*n*) Jun–Sep 2020	Expected value^3^ Jun–Sep 2020						
All trials	US		338.5 (*n* = 1, 354)	351.7	0.96	0.90, 1.03	.25	0.87	0.80, 0.95	< .001
	Non-US		645.0 (*n* = 2580)	584.8	1.1	1.05, 1.16	< .001			
Oncology	US		201.8 (*n* = 807)	189.4	1.06	0.98, 1.16	.15	0.93	0.83, 1.03	.18
	Non-US		345.8 (*n* = 1, 383)	301.3	1.14	1.07, 1.23	< .001			
Cardiovascular	US		73.3 (*n* = 293)	80.2	0.91	0.80, 1.05	.20	0.88	0.75, 1.03	.11
	Non-US		202.0 (*n* = 808)	193.9	1.04	0.96, 1.13	.35			
Mental health	US		78.3 (*n* = 313)	94.7	0.83	0.72, 0.94	.005	0.78	0.66, 0.78	.005
	Non-US		119.0 (*n* = 476)	112.5	1.06	0.95, 1.18	.32			

^1^Total counts for each disease exceed the disease-specific counts noted in the text due to the inclusion of multiple disease studies. ^2^Derived from Poisson regression model, adjusting for calendar month using indicator variables, including interaction terms to derive the risk ratios. ^3^Expected (or counterfactual) monthly average number of trial registrations in the absence of the COVID-19 pandemic. Model expected results based on Poisson regression, adjusting for calendar month using indicator variables

A rebound occurred in the reopening phase. For US-based trials, model-estimated monthly activations (338.5/month) from June 2020 to September 2020 were comparable to expected totals had the pandemic not occurred (351.7/month; relative risk = 0.96, 95% CI 0.90 to 1.03, *p* = .25). For non-US-based trials, estimated monthly activations (645.0/month) substantially exceeded the expected total (584.8/month; relative risk = 1.10, 95% CI 1.05 to 1.16, *p* < .001). Taken together, the rebound in US-based trial activations was relatively weaker (relative risk ratio = 0.87, 95% CI 0.80 to 0.95, *p* < .001).

These patterns were generally similar for oncology, cardiovascular, and mental-health trials (Table 1).

In the post-pandemic period, for US-based studies, the number of excess trials comprised only 8.6% of the number lost during the pandemic period; for non-US-based studies, the number of excess trials was 46.7% of the number lost during the pandemic period.

## Discussion

The COVID-19 outbreak was associated with a substantial decrease in new clinical trial activations, especially for US-based trials. Trial activations rebounded after the initial pandemic period (June 2020 through September 2020), though less so for US-based trials. Moreover, through the end of the analysis period, there remains many fewer new trial activations than what would have occurred in the absence of the COVID-19 pandemic, especially for US-based trials.

These findings are consistent with the disproportionate burden of COVID-19 diagnoses and deaths in the USA during the initial outbreak of the pandemic [2]. The reasons for delays or cancelations in trial activations during the initial pandemic period were not identifiable from the data, a study limitation. Regardless, this reduction is exacerbated by trial recruitment challenges for active studies [5]. This reduction also compounds the overall detrimental impact associated with the pandemic due to delays in diagnosis and treatment of new, non-COVID-19 diseases in the USA as well as other countries hard hit by the pandemic, such as in the UK [6, 7].

## Conclusions

This reduction in new trial activations for non-COVID-19 diseases is likely to slow the pace of clinical research and new drug discovery, with long-term negative consequences for patients. An important question for researchers and policy-makers is whether the renewed outbreak period of winter 2020/2021 will have a similarly negative impact on the initiation of new clinical research studies for non-COVID-19 diseases.

## Data Availability

The data used in this analysis are publicly available through the ClinicalTrials.gov web portal.
